# Emotional Intelligence, Belongingness, and Mental Health in College Students

**DOI:** 10.3389/fpsyg.2020.00093

**Published:** 2020-01-31

**Authors:** Robert W. Moeller, Martin Seehuus, Virginia Peisch

**Affiliations:** ^1^Department of Psychology, Middlebury College, Middlebury, VT, United States; ^2^Department of Psychological Science, University of Vermont, Burlington, VT, United States

**Keywords:** mental health, college students, emotional intelligence, belonging, depression, anxiety, stress, rejection

## Abstract

Mental health problems are prevalent amongst today’s college students and psychosocial stress has been identified as a strong contributing factor. Conversely, research has documented that emotional intelligence (EQ) is a protective factor for depression, anxiety and stress (mental health problems). However, the underlying mechanism whereby EQ may support stronger mental health is currently not well understood. This study used regression analyses to examine the hypothesis that belongingness (inclusion, rejection) partially mediates the effects of EQ (attention, clarity, repair) on psychological well-being in a large sample (*N* = 2,094) of undergraduate students. Results supported the mediation hypotheses for all three EQ components and highlighted that the effects of rejection on psychological well-being were particularly strong. In line with prior research, our results indicate that prevention and intervention efforts with college students could explicitly target EQ skills in an effort to reduce perceived rejection and promote student well-being.

## Introduction

### Mental Health Problems

High rates of mental health problems have been documented amongst college students (for a discussion see Auerbach et al., 2016; Xiao et al., 2017). For example, one study reported that 17% of surveyed students met diagnostic criteria for major depressive disorder (Selkie et al., 2015). Using the Depression, Anxiety, Stress Scale (DASS-21) Mahmoud et al. (2012) found 29% of college students had elevated levels of depression, while 27% had elevated anxiety and 24% elevated stress. The elevated rates of depression, anxiety and stress (mental health problems) are also noted in national data such as those from the American College Health Association’s National College Health Assessment (ACHA-NCHA; American College Health Association, 2019). In their survey of undergraduate students, ACHA reports 26% of students reported feeling so depressed in the past 30 days that it was difficult to function, while 43% of students reported feeling overwhelmed by anxiety in the same period of time (American College Health Association, 2019). While recognizing that many factors contribute to the high rates of psychopathology of college students, past research indicates that psychosocial stress is associated with mental health problems (e.g., Dusselier et al., 2005; Drum et al., 2009). The transition to college is associated with the developmental challenge of changes to existing relationships (Hurst et al., 2013) while college students also experience increased exploration in the context of declining social support systems (Conley et al., 2014). Given the close link between psychosocial stress and student mental health, applied work has explicitly targeted psychosocial functioning of college students (e.g., Pratt et al., 2000; Conley et al., 2013).

### Emotional Intelligence

In light of the increasing mental health problems and the influence of psychosocial factors for college students, it has become increasingly important to understand the role of emotional intelligence of college students as researchers and practitioners begin exploring opportunities for interventions. Emotional intelligence (EQ) includes “the abilities to accurately perceive emotions, to access and generate emotions so as to assist thought, to understand emotions and emotional knowledge, and to reflectively regulate emotions” (Mayer et al., 2004, p. 197). The variability in EQ suggests that some individuals are better able to perceive, correctly identify, and regulate emotions than others (Mayer and Salovey, 1997). Various strands of research suggest that higher levels of EQ are associated with various aspects of psychological well-being, including greater levels of subjective well-being (Sánchez-Álvarez et al., 2015), life satisfaction (Extremera and Fernández-Berrocal, 2005), and better mental health (Martins et al., 2010; Ruiz-Aranda et al., 2012). Further, research has also shown that different aspects of EQ are related to an individual’s ability to perform certain tasks, including academic (Parker et al., 2004; Costa and Faria, 2015) and athletic achievement (Perlini and Halverson, 2006). Focusing specifically on undergraduate students, higher levels of interpersonal and intrapersonal intelligence have been linked to greater college retention (Parker et al., 2006) and end-of-year GPA among first-year students (Schutte et al., 1998; Parker et al., 2004).

Moving beyond emotional adaptation and individual competence, EQ also appears to be involved in the shaping of social functioning. In a study of undergraduate students, researchers found that participants’ EQ was related to their satisfaction with social relationships (Lopes et al., 2003). Specifically, participants who reported having higher levels of emotion regulation abilities were more likely to also report having positive relationships with others, perceiving support from parents, and were less likely to have negative interactions with a friend (Lopes et al., 2003). These results were largely supported by a second study in which an individual’s self-reported emotion regulation abilities were significantly correlated with self-reported positive interactions with friends (Lopes et al., 2004). A noteworthy strength of this study is that the individual’s self-reported emotion regulation abilities were also significantly correlated with friends’ reports of interpersonal functioning (Lopes et al., 2004). Research has demonstrated that higher scores of EQ are associated with more social acceptance and fewer experiences of rejection (Kokkinos and Kipritsi, 2012), as well as larger and more fulfilling social support networks (Ciarrochi et al., 2001). Taken together, these results support the view that the multiple aspects of EQ are associated with better social functioning. Stated differently, individuals who are better able to recognize and regulate their own emotions appear better able to establish and maintain healthy social relationships with peers and parents.

### Sense of Belonging

An important aspect of social functioning is a sense of belonging. The role of perceived belongingness in psychological well-being has also been explored. The seminal work of Baumeister and Leary (1995) provides a valuable theoretical background for this notion. According to the Need to Belong Theory (NBT; Baumeister and Leary, 1995), human beings are motivated to establish a certain amount of stable and positive interpersonal relationships (Baumeister and Leary, 1995). There is extensive evidence to support the NBT. There is a strong positive relation between an individual’s sense of interpersonal belonging and their ratings of happiness and subjective well-being (McAdams and Bryant, 1987). While a lack of social bonds, or explicit feelings of social exclusion, contribute to feelings of anxiety (Baumeister and Tice, 1990; Leary, 1990; Williamson et al., 2018), other mental health outcomes, including depression, loneliness, and social anxiety, are greatly reduced when college students experience a sense of belonging (O’Keeffe, 2013; Stebleton et al., 2014; Raymond and Sheppard, 2018). The need to belong may be particularly pronounced in college students and appears to serve a protective function when satisfied. Yet, despite evidence that EQ is associated with higher quality social interactions with peers (Brackett et al., 2004; Lopes et al., 2004), the relation between EQ and belongingness among college students is not well understood.

### The Current Study

High rates of mental health problems are well documented in today’s college population. In an effort to support the well-being of undergraduate students, predictors of mental health problems need to be identified and fostered. In recognizing that psychosocial stressors are contributing to some of the psychological distress reported by college students, aspects of EQ and belongingness have emerged as correlates of mental health problems. To our knowledge, no study to date has examined the association between the different aspects of EQ, belongingness, and mental health in college students. Additionally, elucidating the effects of the EQ subscales (attention, clarity, repair) on mental health in college students could provide an opportunity to direct interventions that target specific emotional skills. Given that greater levels of each of the aspects of EQ have been associated with better interpersonal relationships, this study tested the hypothesis that belongingness (whether measured as level of acceptance, rejection, or both) mediates the effects of the EQ subscales (attention, clarity, repair) on psychological well-being.

## Materials and Methods

### Procedure

The Middlebury Institutional Review Board (IRB) approved all study procedures. An ongoing longitudinal study, the College Student Mental Health Pathways study, is a study exploring social/emotional development and mental health outcomes among undergraduate college students. The present analysis utilizes data from wave two, collected in 2019. All students at two liberal arts colleges in the United States received an email inviting them to participate in a study about student stress and mental health. Students who clicked on the link in the email were directed to an informed consent page, approved by the primary author’s IRB. Students were able to consent after reading the consent form by selecting one of two radio buttons, ‘I consent to participate’ or ‘I do not consent to participate’. A total of 2,094 students completed wave two of the study, which resulted in a participation rate of 45.86%. At the completion of the survey, students could enter their contact information into a separate survey to participate in a raffle to win a gift card (values ranged from $25–100).

### Measures

#### Demographics

Participants reported demographic information including gender, race/ethnicity, perceived socioeconomic status (SES), and sexual orientation. A majority of the sample identified as female (58.31%, *n* = 1,221), 38.73% (*n* = 811) identified as male and 2.96% (*n* = 62) non-binary. The majority of respondents identified as heterosexual, 79.04% (*n* = 1,655), while 4.06% (*n* = 85) identified as gay/lesbian, and 8.26% (*n* = 173) identified as bisexual. Seventy-three percent (*n* = 1,519) of the sample identified as White, followed by Asian 9.31% (*n* = 195), Latinx 9.03% (*n* = 189), and those identifying as mixed race or other 4.78% (*n* = 100). Perceived SES status included 51.21% (*n* = 1,060) of participants identifying as middle SES, 37.25% (*n* = 771) as high SES, and 11.5% (*n* = 239) as lower SES. The average age of the students was 19.94 (*SD* = 1.33). Demographics are presented in Table 1.

**TABLE 1:** Participant characteristics.

			**Age**	
**Participant characteristics**	***n***	**%**	***M***	***SD***
**All participants**	2,071	100	19.94	1.33
**Gender**				
Female	1,221	58.31	19.94	1.34
Male	811	38.73	19.91	20.62
Other	62	2.96	20.62	1.57
**Sexual Orientation**				
Heterosexual	1,655	79.04	19.93	1.31
Gay/Lesbian	85	4.06	19.96	1.43
Bisexual	173	8.26	19.9	1.36
Other	181	8.64	20.05	1.44
**Race/Ethnicity**				
White	1,519	72.54	19.99	1.33
Asian	195	9.31	19.80	1.29
Black/African American	91	4.35	19.79	1.43
Latinx	189	9.03	19.69	1.36
Other	100	4.78	20.00	1.28
**SES**				
Lower	239	11.55	19.92	1.40
Middle	1,060	51.21	19.85	1.32
High	771	37.25	20.06	1.31

#### Depression, Anxiety, and Stress

The DASS-21 scale (Henry and Crawford, 2005) was used to assess depression, anxiety, and stress. The scale can be utilized as a sum score or as three individual scales (i.e., depression, anxiety, stress). Participants were asked to respond to statements indicating how frequently in the past week they experienced any of the symptoms. Response sets and associated values for scoring were as follow: (0) did not apply to me at all, (1) applied to me to some degree, or some of the time, (2) applied to me a considerable degree or a good part of time, (3) applied to me very much or most of the time. Each scale contained seven items, with associated scores ranging from 0 to 21. Items in the measure include: “I found it difficult to work up the initiative to do things” (depression), “I felt I was close to panic” (anxiety) and “I found it hard to wind down” (stress). Due to the strong intercorrelations between depression, anxiety and stress (see Table 2), the composite DASS score was used to better capture the totality of the mental health experience. Cronbach’s alpha for the full scale was 0.93.

**TABLE 2:** Correlations and descriptive statistics for variables of interest.

**Measures**	**1**	**2**	**3**	**4**	**5**	**6**	**7**	**8**	**9**
**DASS**									
1. Anxiety		0.64***	0.74***	0.88***	−0.36***	0.44***	−0.05*	−0.32***	−0.34***
2. Depression			0.67***	0.88***	−0.51***	0.60***	−0.10***	−0.38***	−0.57***
3. Stress				0.91***	−0.33***	0.44***	0.02	−0.34***	−0.40***
4. Total					−0.45***	0.56***	−0.05*	−0.39***	−0.50***
**GBS**									
5. Inclusion						−0.72***	0.20***	0.33***	0.48***
6. Rejection							−0.16***	−0.39***	−0.53***
**TMMS**									
7. Attention								0.26***	0.22***
8. Clarity									0.37***
9. Repair									
M	7.17	8.72	10.87	26.76	33.50	16.19	67.11	45.20	29.92
SD	7.64	9.00	8.56	22.42	6.36	15.00	10.92	9.53	6.48

*p < 0.05; **p < 0.01; ***p < 0.001.

#### Belongingness

The General Belongingness Scale (GBS; Malone et al., 2012) was used to measure experiences of belongingness. The GBS contains two subscales: Inclusion and Rejection. Each subscale contains six items and participants responded to each item using a 7-point Likert scale ranging from strongly disagree to strongly agree. Sample items include: “I feel accepted by others” (Inclusion) and “When I am with other people, I feel like a stranger” (Rejection). Inclusion and Rejection are potentially orthogonal; it is possible for a respondent to be high (or low) on both, reflecting the simultaneous experience of being included in some circumstances and rejected in others. Cronbach’s alphas were 0.92 for the Inclusion subscale and.89 for the Rejection subscale.

#### Emotional Intelligence

The Trait Meta Mood Scale (TMMS; Salovey et al., 1995) was used to measure three forms of emotional intelligence: attention to emotions (Attention), emotional clarity (Clarity) and repair of emotions (Repair). The TMMS includes 30 items, 13 for Attention, 11 for Clarity, and 6 for Repair. Participants were asked to use a five-point Likert scale (strongly disagree to strongly agree) to indicate their agreement with each item. Example items include: “I pay a lot of attention to how I feel” (Attention), “Sometimes I can’t tell what my feelings are” (Clarity), and “I try to think good thoughts no matter how badly I feel” (Repair). Cronbach’s alphas for the subscales were: 0.87 for Attention, 0.86 for Clarity, and 0.81 for Repair.

#### Statistical Procedures

Three parallel mediation models were independently estimated using the PROCESS macro (Hayes, 2017), using pre-defined Model 4. Consistent with the original conceptualization of the TMMS as consisting of independent subscales (Attention, Clarity, and Repair), and with more recent factor analyses that found low levels of cross-loading amongst empirically observed factors (Palmer et al., 2003), the models were estimated separately in order to illustrate the independent contributions of each subscale. Models were estimated both with and without demographic covariates. Covariates tested were gender identification, socioeconomic status, sexual orientation, and race/ethnicity, all dummy coded to allow for their inclusion in ordinary least squares regression modeling. The resulting models including covariates did not differ in significance, sign, or approximate coefficient value from the models that did not include covariates. For ease of interpretation the models represented do not show the covariates.

## Results

Bivariate correlations were estimated for variables of interest and are shown in Table 2. Note that statistically significant (and meaningfully large) correlations were observed amongst most of the variables, with only the relationships between Attention and Stress having a *p* > 0.05, and only the relationships between Stress and Anxiety and the DASS Full Scale having an estimated *p* > 0.01. The correlations between the DASS Full Scale and the DASS subscales are presented for completeness, but should be interpreted with caution, since the full scale consists of the sum of the subscales, and thus the measures are not independent.

Tables 3–6 show differences in the variables of interest by gender (Table 3), socioeconomic status (Table 4), sexual orientation (Table 5), and race/ethnicity (Table 6). Significance was calculated using ANOVAs, and is marked with subscripts on all three tables at the *p* < 0.05 level.

**TABLE 3:** Gender differences in Depression Anxiety Stress Scale (DASS), Trait Meta Mood Scale (TMMS) and General Belongingness Scale (GBS).

	**Man**		**Woman**		**Other**	
	***M***	***SD***	***M***	***SD***	***M***	***SD***
**DASS**						
Full scale	23.51a	21.58	28.52b	22.36	40.88c	26.41
Anxiety	6.21a	7.17	7.72b	7.80	10.81c	8.88
Depression	8.24a	8.77	8.86a	8.95	14.44b	11.36
Stress	9.05a	8.08	11.94b	8.56	15.62c	9.84
**TMMS**						
Attention	64.08a	11.10	68.82b	10.53	67.71a,b	11.96
Clarity	45.91a	9.50	44.76b	9.54	43.58a,b	9.78
Repair	30.00a	6.20	29.88a	6.62	26.12b	6.66
**GBS**						
Inclusion	33.33a	6.41	33.66a	6.36	30.22b	7.08
Rejection	16.18a	7.47	16.08a	7.47	20.72b	8.54

**Values in the same now with a different subscript are significantly different at p < 0.05.**

**TABLE 4:** Socioeconomic differences in Depression Anxiety Stress Scale (DASS), Trait Meta Mood Scale (TMMS), and General Belongingness Scale (GBS).

	**Lower**		**Middle**		**Upper**	
	***M***	***SD***	***M***	***SD***	***M***	***SD***
**DASS**						
Full scale	31.20a	24.76	26.54b	22.38	25.71b	21.34
Anxiety	8.60a	8.63	7.16b	7.67	6.78b	7.20
Depression	10.76a	9.77	8.79b	9.04	7.99b	8.49
Stress	11.85a	9.20	10.60b	8.35	10.94a,b	8.56
**TMMS**						
Attention	65.09a	11.69	66.85b	10.94	67.64b	10.87
Clarity	43.38a	10.19	45.14b	9.42	45.82b	9.44
Repair	28.58a	6.84	29.74b	6.35	30.41c	6.49
**GBS**						
Inclusion	31.16a	7.15	33.19b	6.45	34.59c	5.85
Rejection	19.67a	7.84	16.37b	7.4	14.87c	7.17

**Values in the same now with a different subscript are significantly different at p < 0.05.**

**TABLE 5:** Sexual orientation differences in Depression Anxiety Stress Scale (DASS), Trait Meta Mood Scale (TMMS), and General Belongingness Scale (GBS).

	**Heterosexual**		**Gay/lesbian**		**Bisexual**		**Other**	
	***M***	***SD***	***M***	***SD***	***M***	***SD***	***M***	***SD***
**DASS**								
Full scale	24.78a	21.45	35.92b	25.98	36.44b	25.29	32.07b	21.13
Anxiety	6.65a	7.32	10.05b,c	9.86	9.96b	8.51	8.19c	7.41
Depression	8.05a	8.55	11.85b	10.39	11.93b	11.05	10.41b	8.54
Stress	10.08a	8.30	14.03b	9.77	14.55b	8.42	13.47b	8.52
**TMMS**								
Attention	66.34a	10.82	66.81a	13.27	69.91b	11.31	70.25b	10.46
Clarity	45.53a	9.43	44.04a,b	9.44	43.40b	10.45	44.30a,b	9.52
Repair	30.31a	6.31	27.85b	7.51	28.16b	7.42	28.12b	5.67
**GBS**								
Inclusion	33.88a	6.24	31.66b	6.66	31.96b	7.36	31.80b	6.22
Rejection	15.50a	7.25	19.80b	8.2	19.02b	8.27	18.39b	7.26

**Values in the same now with a different subscript are significantly different at p < 0.05.**

**TABLE 6:** Racial/ethnic differences in Depression Anxiety Stress Scale (DASS), Trait Meta Mood Scale (TMMS), and General Belongingness Scale (GBS).

	**Asian**		**Black**		**Hispanic**		**White**	
	***M***	***SD***	***M***	***SD***	***M***	***SD***	***M***	***SD***
**DASS**								
Full scale	28.67a,b	22.44	26.35a,b	23.11	30.40a	24.82	25.93b	21.76
Anxiety	7.63a,b	7.36	7.58a,b	7.88	8.61a	8.49	6.90b	7.47
Depression	10.31a	9.40	8.82a,b	8.93	10.20a	10.02	8.26b	8.68
Stress	10.74a	8.22	9.95a	8.65	11.60a	9.06	10.77a	8.44
**TMMS**								
Attention	63.48a	11.40	65.38a,c	10.09	64.75a,c	12.33	67.77b	10.69
Clarity	44.04a	9.11	45.70a	9.43	44.48a	10.55	45.38a	9.51
Repair	28.53a	7.02	29.71a,b	6.66	29.20a,b	6.60	30.20b	6.37
**GBS**								
Inclusion	31.79a	6.54	32.50a	6.23	32.02a	6.83	34.01b	6.26
Rejection	18.68a	7.80	18.09a	7.30	17.25a	7.41	15.55b	7.37

**Values in the same now with a different subscript are significantly different at p < 0.05.**

**FIGURE 1:**
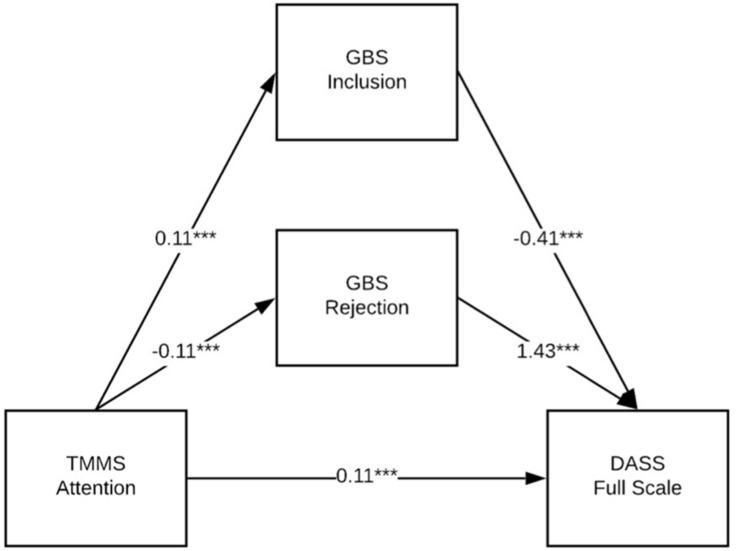
GBS Inclusion and Rejection partially mediate the relationship between TMMS Attention and DASS Full scale. *p < 0.05; **p < 0.01; ***p < 0.001.

**FIGURE 2:**
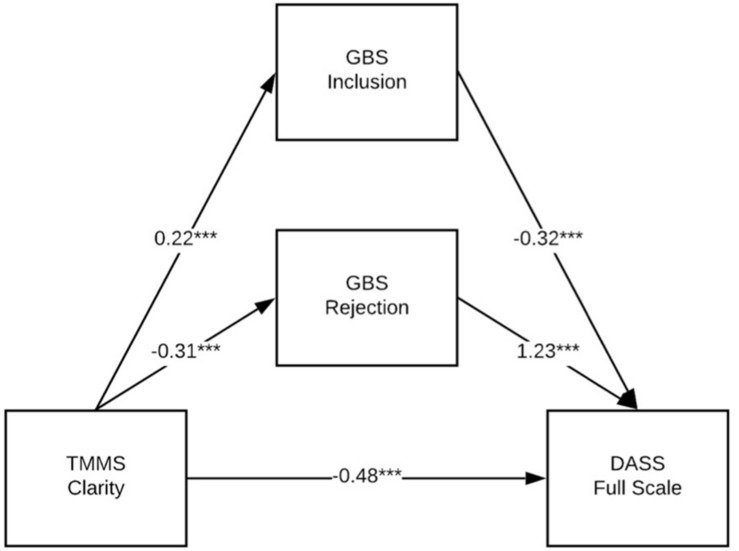
GBS Inclusion and Rejection partially mediate the relationship between TMMS Clarity and DASS Full scale. *p < 0.05; **p < 0.01; ***p < 0.001.

**FIGURE 3:**
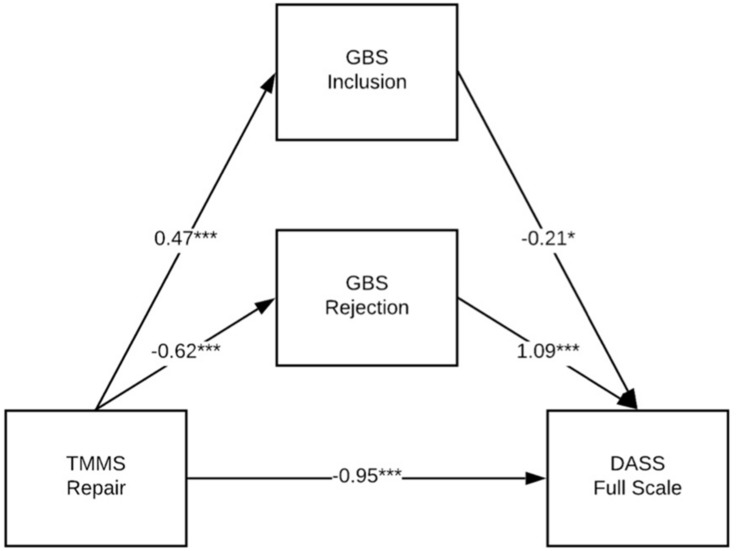
GBS Inclusion and Rejection partially mediate the relationship between TMMS Repair and DASS Full scale. *p < 0.05; **p< 0.01; ***p < 0.001.

Tables 7–9 show the results of a series of parallel mediation models conducted with PROCESS (Hayes, 2017). These models tested whether the relationship between each of the three TMMS subscales (Attention, Clarity, and Repair) and the DASS Full Scale measure of mental health symptoms was mediated by either or both of the GBS scales (Inclusion and Rejection). Thus, Model 1 (see Figure 1 for an illustration and Table 7 for details) tests whether the relationship between Attention and the DASS Full Scale is mediated by Inclusion, Rejection or both; Table 8 and Figure 2 show the same model, but with Clarity; and Table 9 and Figure 3 show the same model, but with Repair. Both the unstandardized and fully standardized coefficients are presented for the total effect of each indirect path, for each model. As per Hayes (2017), the fully standardized coefficients are reasonable measures of effect size, although some debate persists about how best to present effect sizes for more complex mediation models. The standardized coefficients for each indirect path represent the predicted change in DASS Full Scale (as measured in standard deviations) associated with a one standard deviation change in TMMS Attention, Clarity, or Repair (respectively).

**TABLE 7:** Parallel mediation model of TMMS Attention predicting DASS Full scale, mediated by GBS Inclusion and Rejection.

**Outcome variable**	**Predictor variable**	**Coefficient**	**SE**	**p**	**95% CI**
**Direct Effects**					
GBS Inclusion	*F*(1, 1914) = 73.70, *p* < 0.001; *r*^2^ = 0.04				
	Constant	26.06	0.88		
	TMSS Attention	0.11	0.01	< 0.001	[0.09, 0.14]
GBS Rejection	*F*(1, 1914) = 50.27, *p* < 0.001; *r*^2^ = 0.03				
	Constant	23.52	1.04		
	TMMS Attention	−0.11	0.02	< 0.001	[−0.14, −0.08]
DASS Full scale	*F*(3, 1912) = 296.29, *p* < 0.001; *r*^2^ = 0.32				
	Constant	10.63	4.85		
	TMMS Attention	0.11	0.04	< 0.001	[0.03, 0.18]
	GBS Inclusion	−0.41	0.10	< 0.001	[−0.60, −0.22]
	GBS Rejection	1.43	0.08	< 0.001	[1.27, 1.59]
**Total Effect Model**					
DASS Full scale	*F*(1, 1914) = 4.60, *p* = 0.03; *r*^2^ = 0.002				
	Constant	33.43	3.15		
	TMMS Attention	−0.10	0.05	0.03	[−0.19, −0.01]
Total effect of TMMS Attention on DASS Full scale		−0.10	0.05	0.03	[−0.19, −0.01]
Direct effect of TMMS Attention on DASS Full scale		0.11	0.04	0.009	[0.03,0.18]
Indirect effects of TMMS Attention on DASS Full scale					
Total indirect effect [standardized coefficient]		−0.20 [−0.10]	0.03		[−0.26, −0.15]
Through GBS Inclusion [standardized coefficient]		−0.05 [−0.02]	0.01		[−0.08, −0.02)
Through GBS Rejection [standardized coefficient]		−0.16 [−0.08]	0.02		[−0.20, −0.11]

**TABLE 8:** Parallel mediation model of TMMS Clarity predicting DASS Full scale, mediated by GBS Inclusion and Rejection.

**Outcome variable**	**Predictor variable**	**Coefficient**	**SE**	***p***	**95% CI**
**Direct Effects**					
GBS Inclusion	*F*(1, 1904) = 233.49, *p* < 0.001; *r*^2^ = 0.11				
	Constant	23.50	0.67		
	TMSS Clarity	0.22	0.01	<0.001	[0.19, 0.25]
GBS Rejection	*F*(1, 1904) = 341.72, *p* < 0.001; *r*^2^ = 0.15				
	Constant	30.07	0.77		
	TMSS Clarity	−0.31	0.02	<0.001	[−0.34, −0.27]
DASS Full scale	*F*(3, 1902) = 341.01, *p* < 0.001; *r*^2^ = 0.35				
	Constant	39.09	4.74		
	TMSS Clarity	−0.48	0.05	<0.001	[−0.57, −0.38]
	GBS Inclusion	−0.32	0.09	<0.001	[−0.50, −0.13]
	GBS Rejection	1.23	0.08	<0.001	[1.07, 1.39]
**Total Effect Model**					
DASS Full scale	*F*(1, 1904) = 349.28, *p* < 0.001; *r*^2^ = 0.16				
	Constant	68.59	2.29		
	TMMS Clarity	−0.92	0.05	<0.001	[−1.02, −0.83]
Total effect of TMMS Clarity on DASS Full scale		−0.92	0.05	<0.001	[−1.02, −0.83]
Direct effect of TMMS Clarity on DASS Full scale		−0.48	0.47	<0.001	[−0.57, −0.38]
Indirect effects of TMMS Clarity on DASS Full scale					
Total indirect effect [standardized coefficient]		−0.45 [−0.19]	0.03		[−0.51, −0.39]
Through GBS Inclusion [standardized coefficient]		−0.07 [−0.03]	0.03		[−0.12, −0.02]
Through GBS Rejection [standardized coefficient]		−0.38 [−0.16]	0.04		[−0.45, −0.31]

**TABLE 9:** Parallel mediation model of TMMS Repair predicting DASS Full scale, mediated by GBS Inclusion and Rejection.

**Outcome variable**	**Predictor variable**	**Coefficient**	**SE**	***p***	**95% CI**
**Direct Effects**					
GBS Inclusion	*F*(1, 1922) = 581.69, *p* < 0.001; *r*^2^ = 0.23				
	Constant	19.30	0.60		
	TMMS Repair	0.47	0.02	<0.001	[0.44, 0.51]
GBS Rejection	*F*(1, 1922) = 756.73, *p* < 0.001; *r*^2^ = 0.29				
	Constant	34.61	0.68		
	TMMS Repair	−0.62	0.02	<0.001	[−0.66, −0.57]
DASS Full scale	*F*(3, 1920) = 370.50, *p* < 0.001; *r*^2^ = 0.37				
	Constant	44.27	4.62		
	TMSS Repair	−0.95	0.08	<0.001	[−1.09, −0.80]
	GBS Inclusion	−0.21	0.09	0.03	[−0.39, −0.02]
	GBS Rejection	1.09	0.08	<0.001	[0.93, 1.25]
**Total Effect Model**					
DASS Full scale	*F*(1, 1922) = 631.20, *p* < 0.001; *r*^2^ = 0.25				
	Constant	78.08	2.09		
	TMMS Repair	−1.72	0.07	<0.001	[−1.85, −1.58]
Total effect of TMMS Repair on DASS Full scale		−1.72	0.07	<0.001	[−1.85, −1.58]
Direct effect of TMMS Repair on DASS Full scale		−0.95	0.08	<0.001	[−1.09, −0.80]
Indirect effects of TMMS Repair on DASS Full scale					
Total indirect effect [standardized coefficient]		−0.77 [−0.22]	0.05		[−0.88, −0.66]
Through GBS Inclusion [standardized coefficient]		−0.10 [−0.03]	0.05		[−0.21, 0.004]
Through GBS Rejection [standardized coefficient]		−0.67 [−0.19]	0.06		[−0.80, −0.55]
					

All three models accounted for a significant portion of the variance in the outcome measure; see Tables 7–9 and Figures 1–3 for coefficients and model fit information. The 95% CI for the indirect path between TMMS Repair and DASS Full Scale through GBS Inclusion included zero, which suggests that the strength of that pathway is not of meaningful or statistically significant size. Note that all models reflect partial mediation, and that a protective indirect effect of Attention (through Inclusion and Rejection) is partially suppressed by a deleterious direct effect of Attention of mental health burden. Note that the size of this sample may reduce the interpretability of NHST measures of significance, and that the size and sign of the coefficients are more meaningful.

## Discussion

This study sought to elucidate the association between EQ and adaptive functioning in college students. Specifically, the models tested whether sense of belongingness mediates the association between EQ and adaptation. We hypothesized that students with stronger EQ abilities would report higher levels of belongingness which, in turn, would be associated with better mental health. Conversely, we also expected that students with lower levels of EQ would be more likely to experience rejection which, in turn, would be linked to higher levels of depression, anxiety, and stress.

These results broadly supported our hypothesis: students with more EQ (as evidenced by higher scores on any or all of the subscales) experienced higher levels of belongingness (more inclusion and less rejection) which, in turn, was associated with lower overall mental health problems. The exception was the indirect pathway between TMMS Repair and DASS Full Scale through GBS Inclusion, which was not of meaningful size. While inclusion was found to be meaningful in predicting mental health, it was the experience of rejection that was the stronger predictor of mental health outcomes. Specifically, students with lower levels of EQ are experiencing higher levels of rejection, and it is rejection which has the most significant impact on the DASS full scale mental health outcome. These results implicitly support the modeling of inclusion and rejection as orthogonal scales, as per the GBS (Malone et al., 2012). The effects of rejection on depression in adolescent populations is well established (for a review see Platt et al., 2013). Our findings extend the existing research by demonstrating that among emerging adults, the experience of rejection is associated with higher levels of mental health problems. The experience of being included does have a protective effect, but, since high levels of inclusion and rejection can be experienced by the same person, working to improve inclusion is unlikely to be sufficient to reduce mental health burdens: the reduction of experience of rejection is likely to have a larger impact.

### Implications

These findings have implications for applied work. Results from our mediation analyses suggested a strong link between perceived rejection and mental health problems. Such results tentatively suggest that intervention efforts could target students who are experiencing feelings of rejection or isolation within their college community. Once identified, these students could be targeted with additional supports, such as short-term counseling, to support well-being. Taking a preventative approach, campus initiatives that support regular and healthy student interactions should continue to receive funding such that they can be maximally effective. A focus on increasing students’ sense of belonging should also seek to lower experiences of rejection. Given that each of the scales of EQ was independently related to sense of belongingness, targeting and strengthening emotional intelligence would also be a potential avenue for prevention and intervention efforts. However, further research is needed to further elucidate the association between EQ, belongingness, and mental health in college samples. Such research should address both the differences in impact between the EQ subscales and explore the extent to which Attention, Clarity, and Repair may vary in their malleability. If, as these results suggest, they are each independently linked to important mental health outcomes, then a targeted intervention would be most effective if it targeted the aspect of EQ most susceptible to intentional change.

### Limitations

Our results should be interpreted in the context of the study’s limitations. First, the study was based on student self-report, which has inherent and well-documented limitations. A second weakness relates to the representativeness of our sample; participants were recruited from two small, competitive liberal arts colleges thereby potentially limiting generalizability of study findings. Similarly, there might be systematic differences between those students who decided to complete the survey and those who chose not to participate. Lastly, data was collected at one timepoint, which limits our ability to make strong inferences about causality. Future research should recruit samples that are more representative of the overall college student population and consider using multi-informant assessments (e.g., friends, parents) to corroborate the self-report data. Longitudinal data collection could also help establish the causal relationship between the three study variables. These limitations notwithstanding, our findings expand what is known about college student well-being by suggesting that EQ and a sense of belongingness are related to mental health symptoms of college students.

## Data Availability Statement

The datasets generated for this study will not be made publicly available in order to maintain confidentiality of the study participants. Requests to access the datasets should be directed to the corresponding author.

## Ethics Statement

The studies involving human participants were reviewed and approved by the Middlebury College Institutional Review Board. The patients/participants provided their electronic informed consent to participate in this study.

## Author Contributions

RM and MS contributed conception, design, and database organization. RM, MS, and VP contributed equally to analyses, draft of the manuscript as well as revisions, and approved the submitted version.

## Conflict of Interest

The authors declare that the research was conducted in the absence of any commercial or financial relationships that could be construed as a potential conflict of interest.
